# 

**Published:** 1838-10

**Authors:** 


### CONTENTS OF No. XII.

OF THE

### British and Foreign Medical Review.

SEPTEMBER, 1838.

---

### Part First.—Analytical and Critical Reviews.

PAGE

Art. I.—1. Influence de l'Anatomie Pathologique sur la Médecine, depuis Morgagni jusqu a nos Jours. Par Risueno D'Amador . . . 293

Influence of Pathological Anatomy upon Medicine, from the time of Morgagni to the present day. By Professor Risueno D'Amador.

2. Mémoire en Réponse à cette Question : "Quelle a été l'Influence de l'Anatomie Pathologique sur la Médecine, depuis Morgagni jusqu'à nos Jours?" Par Constant Saucerotte, m.d. . . . . . ib.

Memoir in Reply to the Question: "What has been the Influence of Pathological Anatomy upon Medicine, from the time of Morgagni to the present day?" By Constant Saucerotte, m.d.

Art. II.—1. Remarks on the Treatment of Fractures of the lower Extremities without the aid of Splints. By J. F. Burke, m.r.c.s. . . 308

2. A Practical Treatise on Fractures. By Edward F. Lonsdale, Surgeon . ib.

Art. III.—1. Commentatio de Delirio Tremente. Auct. Dr. Höegh-Guldberg 320

An Essay on Delirium Tremens. By O. C. Höegh-Guldberg, m.d.

2. Prize Dissertation (for 1831) on Delirium Tremens. By J. C. Cross, m.d. . ib.

Art. IV.—An Experimental Enquiry into the Physiology of Cutaneous Absorption, and its Application to Therapeutics, &c. By W. H. Madden, m.d. . 332

Art. V.—1. Final Report of the Committee of the Philadelphia Medical Society, on the Construction of Instruments, and their Mode of Action, in the radical Cure of Hernia, &c. By Heber Chase, m.d. . . . 341

2. De la Cure Radicale des Hernies. Par M. le Docteur Belmas . . ib.

3. Uber radicalen Heilung reponibler Brüche. Von Dr. Ph. Finck . . ib.

Art. VI.—Nouvelles Recherches sur le Rheumatisme articulaire aigu en général, &c. Par Professeur J. Bouillaud . . . . 352

New Researches on acute and articular Rheumatism, &c. By J. Bouillaud.

2. Leçons de Clinique Médicale faites à l'Hôtel-Dieu de Paris. Par le Professeur A.F. Chomel. (Rheumatisme et Goutte.) . . . ib.

Lectures on Clinical Medicine, delivered at the Hôtel-Dieu, Paris. By Prof. A. F. Chomel. (Rheumatism and Gout.)

5

PAGE

ART. VII.—The Transactions of the Provincial Medical and Surgical Association. Vol. VI. . . . . . . . 380

Retrospective Address. By Dr. BARDSLEY . . . . ib.

Plan for the Reports of Infirmaries and Dispensaries. By Dr. COWAN . 381

On the Medical Topography of Exeter. By Dr. SHAPTER . . 382

On the Medical Topography and Statistics of Cheltenham. By Mr. NASH . 384

Cursory Examination of the Works of Galen. By Dr. KIDD . . 385

On the Treatment of Hypertrophy of the Heart, &c. By Mr. SALTER . 387

Two Cases of Gangrene of the Lungs. By Dr. ENGLAND . . 390

Case of partial Ectopia Cordis and Umbilical Hernia. By Dr. O'BRYEN . 391

Extirpation of the Eye. By Mr. MIDDLEMORE . . . ib.

Reports of Out-Patients treated in the Birmingham-Town Infirmary. By Mr. T. RYLAND and Mr. S. BERRY . . . . ib.

Report of Cases treated at the Birmingham Dispensary. By Dr. O. WARD . ib.

Report of Cases at the Birmingham Eye Infirmary. By Mr. MIDDLEMORE . 392

Account of the In-Patients in the Geneva Hospital. By Dr. LOMBARD . 393

Influenza or Epidemic Catarrh of the Winter, 1836-7. By Dr. STREETEN . ib.

ART. VIII.—1. Beobachtung einer auffallenden bisher unerkannten Structur des Seelenorgans bei Menschen und Thieren. Von C. G. EHRENBERG . ib.

Discovery of a remarkable Structure of the Brain and Nerves in Man and Animals. By C. G. EHRENBERG.

2. Anatomie der mikroskopischen Gebilde des menschlichen Körpers. Von Dr. JOSEPH BERRES . . . . . . 394

Microscopical Anatomy of the Tissues of the Human Body. By Dr. BERRES.

3. Beiträge zur Aufklärung der Erscheinungen und Gesetze des organischen Lebens. Von GOTTFRIED-REINHOLD TREVIRANUS . . . . . ib.

Contributions to illustrate the Phenomena and Laws of Organic Life. By GOTTFRIED-REINHOLD TREVIRANUS.

4. Vorläufige Mittheilung microscopischen Beobachtungen über den innern Bau der Cerebrospinalnerven und über die Entwickelung ihrer Formelemente. Von ROBERT REMAK . . . . . . ib.

Provisional Communication of microscopical Observations on the intimate Structure of the Cerebro-Spinal Nerves, &c. By ROBERT REMAK.

5. Ueber den Verlauf und die letzten Enden der Nerven. Von G. VALENTIN . ib.

On the Course and Terminations of the Nerves. By G. VALENTIN.

6. Ueber die Endigungsweise der Nerven in den Muskeln, nach eigenen Untersuchungen. Von Dr. FRIEDRICH-CARL EMMERT . . . ib.

On the Mode of Termination of the Nerves in the Muscles. By Dr. EMMERT.

7. Beitrag zur mikroskopischen Anatomie der Nerven. Von Dr. ERNST BURDACH ib.

Contribution to the Microscopical Anatomy of the Nerves. By Dr. BURDACH.

8. Elements of Physiology. By J. MUELLER, M.D. Translated from the German, with Notes, by WM. BALY, M.D. . . . . ib.

ART. IX.—1. Practical Remarks on the Diseases of the Skin, on the external Signs of Disorder, and on the Constitutional Peculiarities during Infancy and Childhood. By WALTER C. DENDY, Surgeon . . . 425

2. A short Treatise on the external Characters, Nature, and Treatment of the different Forms of Porrigo, or Scalled Head, and Ringworm. By WALTER DICK, M.D. . . . . . . . ib.

PAGE

ART. X.—1. Etudes Chimiques sur le Sang Humain. Par Dr. LOUIS-RENÉ LE CANU 431

Chemical Researches on the Human Blood. By L. R. LE CANU, M.D.

2. Nouvelles Expériences sur le Sang. Par M. P. DENIS . . . ib.
New Experiments on the Blood. By M. P. DENIS.

ART. XI.—Notes on the Medical History and Statistics of the British Legion of Spain; comprising the Results of Gun-shot Wounds, in relation to important Questions in Surgery. By RUTHERFORD ALCOCK, K.T.S. &c. . 448

ART. XII.—1. Inaugural Dissertation on the Presence of Air in the Organs of Circulation. By JOHN ROSE CORMACK . . . . 455

2. Remarks on a Case of Suicide, published by Dr. P. D. HANDYSIDE, in No. 134 of the Edinburgh Medical and Surgical Journal. By J. R. CORMACK, M.D. ib.

3. Bulletin de l'Académie Royale de Médecine. Tome II. No. 12. Rapport de M. BOUILLAUD sur les Expériences relatives à l'Introduction de l'Air dans les Veines, faites par M. AMUSSAT.—Discussion sur l'Introduction de l'Air dans les Veines. Ib. . . . . . . 456

4. Lettre sur l'Introduction de l'Air dans les Veines de l'Homme. Par M. VELPEAU ib.

ART. XIII.—The Life of EDWARD JENNER, M.D. LL.D. F.R.S. &c. With Illustrations of his Doctrines, and Selections from his Correspondence. By JOHN BARON, M.D. F.R.S. &c. . . . . . 477

ART. XIV.—Counter-Irritation, its Principles and Practice; illustrated by one hundred Cases of the most painful and important Diseases effectually cured by external Applications. By A. B. GRANVILLE, M.D. F.R.S. &c. . 498

ART. XV.—Practical and Experimental Chemistry, adapted to Arts and Manufactures. By E. MITSCHERLICH, Professor of Chemistry, &c. Translated from the first Portion of his Compendium, by STEPHEN LOVE HAMMICK, M.D. . 505

ART. XVI.—An Essay on the Antiquity of Hindoo Medicine, including an Introductory Lecture to the Course of Materia Medica and Therapeutics delivered at King's College. By J. F. ROYLE, M.D. F.R. and L.S. &c. . . 506

---

### PART SECOND.—Bibliographical Notices.

ART. I.—On Diseases of the Bladder. By WM. COULSON, Surgeon . . 509

ART. II.—Manual of British Botany. By D. C. MACREIGHT, M.D. . . 511

ART. III.—The Narrative of a Recovery from Tic Douloureux. By the Rev. C. E. HUTCHINSON . . . . . . . ib.

ART. IV.—Practical Observations on Hysteria, especially relating to its Organic Character. By JOHN PRICHARD, M.R.C.S. . . . . 512

ART. V.—Lehrbuch der vergleichenden Physiologie der Haus-Saügethiere. Von Dr. E. F. GURLT . . . . . . . 513

Elements of the Comparative Physiology of the Domestic Mammalia. By Dr. E. F. GURLT.

PAGE

Art. VI.—Die Nachtheile unzeitiger und uebermässiger Anwendung des Aderlasses und anderer Blutentziehung. Von Dr. L. Wetzlar . . 515

On the injurious Consequences of unnecessary and immoderate Bloodletting. By Dr. Wetzlar.

Art. VII.—A Practical Compendium of the Materia Medica; with numerous Formulæ adapted for the Treatment of the Diseases of Infancy and Childhood. By Alexander Ure, m.d. . . . . 516

---

### Part Third.—Selections from the British and Foreign Journals.

### I. THE FOREIGN JOURNALS.

#### ANATOMY AND PHYSIOLOGY.

Experiments on the Introduction of Air into the Veins, made by M. Amussat, before a Commission of the Royal Academy of Medicine of Paris . 517

On the Influence of Galvanism on the Process of Artificial Digestion. By Drs. Purkinje and Pappenheim . . . . . 527

#### PATHOLOGY, PRACTICAL MEDICINE, AND THERAPEUTICS.

Cauterization of the Pharynx in Croup. By Dr. Felix Hatin . . 530

On the Employment of the Hydrochlorate of Tin. By Dr. Schlessinger . ib.

On Epidemic Epilepsy in Schools. By Dr. Meyer . . . 531

Treatment of Spermatorrhœa . . . . . . 532

On Artificial Auscultation. By M. Petrequin . . . . ib.

On the Use of the Essential Oil of Turpentine in Diseases of the Eye. By Dr. A. Trinchinetti . . . . . . 533

Employment of Chlorine in acute and chronic Bronchitis. By Dr. Toulmouche ib.

Of the Symptoms of the earliest Stage of Phthisis Pulmonalis. By M. Fournet 534

#### SURGERY.

On Chopart's Method of Amputating the Foot. By M. Blandin . . 535

On Rupture of the Perineum in Females. By Professor Dieffenbach . 536

Beneficial Action of Acetate of Lead in incarcerated Hernia. By Dr. Huxthausen 537

On Hemlock Baths in Cutaneous Diseases. By Dr. Fantonetti . . 538

Dislocation of all the Metatarsal Bones upon the Tarsus. By M. Mazet . ib.

#### MIDWIFERY.

On Puerperal Fever. By Dr. A. Gruber . . . . 539

Case of Spontaneous Rupture of the Uterus. By M. Gendrin . . ib.

#### MEDICAL JURISPRUDENCE.

Important Cases of Death after Wounds and Injuries . . . 540

### ANIMAL CHEMISTRY.

PAGE

Chemical Composition of Human Lymph. By R. T. MARCHAND and C. COLBERG 544

Microscopical Researches on the Composition of Vaccine Fluid. By M. DUBOIS 546

Microscopical Examination of Pus, Mucus, and Effused Fluids. By Dr. MANDT ib.

### II. THE AMERICAN AND COLONIAL JOURNALS.

### MEDICINE.

Case of Pneumonia, with Polypiform Concretion in the Heart. By Professor DUNGLISON . . . . . . . 547

### SURGERY.

Operations for the Excision of the Ribs. By Dr. WARREN . . . 548

Two Cases of double Amputation of the Legs from Frost-bite. By Dr. SEWELL . 550

### III. THE BRITISH JOURNALS.

### PHYSIOLOGY.

Observations on the Fluid in the Vesiculæ Seminales of Man. By JOHN DAVY, M.D. 551

An Experimental Enquiry into the Influence of Nitrogen on the Growth of Plants. By ROBERT RIGG, Esq. . . . . . 552

Researches on Suppuration. By GEORGE GULLIVER, Esq. . . . 553

On the Differences of the Laws regulating Vital and Physical Phenomena. By WM. B. CARPENTER, M.R.C.S. . . . . . ib.

### PATHOLOGY, PRACTICAL MEDICINE, AND THERAPEUTICS.

On the State of the Pupil in Typhus, and the use of Belladonna in certain Cases of Fever. By ROBERT J. GRAVES, M.D. . . . . 554

A Statistical Enquiry on Fever. By ARTHUR SAUNDERS THOMSON, M.D. &c. . 556

On the Treatment of Rheumatic Gout. By A. L. WIGAN, Esq. . . ib.

New Mode of exhibiting Copaiva. By CHARLES EVANS, Esq. . . 558

### SURGERY.

On the Primary Causes of Strangulation, and an improved Mode of performing the Taxis in Cases of intestinal Herniæ. By JAMES O'BEIRNE, M.D. . ib.

Observations arising out of the Results of Amputation in different Countries. By BENJAMIN PHILLIPS, Esq. F.R.S. . . . . 561

On the Treatment of Small-pox by puncturing the Pustules. By J. LANGLEY, Esq. 563

On the Efficacy of Pressure in certain Cases of Venereal Phagedænic Ulceration. By HUGH CARMICHAEL, A.M. . . . . . 564

On Chronic Cystitis, &c. By JOHN O'BRYEN, M.D. M.R.C.S. . . . 565

### MIDWIFERY.

PAGE

On the best Means of applying Pressure to the Uterus after Delivery. By J. L. FENNER, Esq. . . . . . . 567

---

### PART FOURTH.—Medical Intelligence.

Provincial Medical and Surgical Association . . . . 569
British Association for the Advancement of Science . . . 573
Medical Relief under the New Poor-law . . . . . 586
New Work on Physiology. By W. B. CARPENTER . . . 590
College of Surgeons: Adjudication of the Jacksonian Prize . . . ib.
Account of a fresh Supply of Vaccine Virus from the Cow. By M. ESTLIN . 591
On the Medical Institutions of Cairo. By CLOT-BEY . . . 592
Prussian Regulations regarding Leeches . . . . 593
OBITUARY.—JOHN SIMS, M.D. . . . . . 594
BOOKS RECEIVED FOR REVIEW . . . . . ib.

INDEX, TITLE, &c.

